# The Interplay Between Reproductive Tract Microbiota and Immunological System in Human Reproduction

**DOI:** 10.3389/fimmu.2020.00378

**Published:** 2020-03-16

**Authors:** Salwan Al-Nasiry, Elena Ambrosino, Melissa Schlaepfer, Servaas A. Morré, Lotte Wieten, Jan Willem Voncken, Marialuigia Spinelli, Martin Mueller, Boris W. Kramer

**Affiliations:** ^1^Department of Obstetrics and Gynecology, GROW School of Oncology and Developmental Biology, Maastricht University Medical Centre (MUMC), Maastricht, Netherlands; ^2^Department of Genetics and Cell Biology, Faculty of Health, Medicine and Life Sciences, Research School GROW (School for Oncology & Developmental Biology), Institute for Public Health Genomics, Maastricht University, Maastricht, Netherlands; ^3^Department of Obstetrics and Gynecology, University Hospital Bern, University of Bern, Bern, Switzerland; ^4^Laboratory of Immunogenetics, Department Medical Microbiology and Infection Control, VU University Medical Center, Amsterdam UMC, Amsterdam, Netherlands; ^5^Tissue Typing Laboratory, Department of Transplantation Immunology, Maastricht University Medical Centre, Maastricht, Netherlands; ^6^Department of Molecular Genetics, Maastricht University Medical Centre, Maastricht, Netherlands; ^7^Department of Pediatrics, Maastricht University Medical Centre, Maastricht, Netherlands

**Keywords:** microbiota, immunology, female reproductive tract, vaginal, endometrial, placental, preterm birth, pregnancy complications

## Abstract

In the last decade, the microbiota, i.e., combined populations of microorganisms living inside and on the surface of the human body, has increasingly attracted attention of researchers in the medical field. Indeed, since the completion of the Human Microbiome Project, insight and interest in the role of microbiota in health and disease, also through study of its combined genomes, the microbiome, has been steadily expanding. One less explored field of microbiome research has been the female reproductive tract. Research mainly from the past decade suggests that microbial communities residing in the reproductive tract represent a large proportion of the female microbial network and appear to be involved in reproductive failure and pregnancy complications. Microbiome research is facing technological and methodological challenges, as detection techniques and analysis methods are far from being standardized. A further hurdle is understanding the complex host-microbiota interaction and the confounding effect of a multitude of constitutional and environmental factors. A key regulator of this interaction is the maternal immune system that, during the peri-conceptional stage and even more so during pregnancy, undergoes considerable modulation. This review aims to summarize the current literature on reproductive tract microbiota describing the composition of microbiota in different anatomical locations (vagina, cervix, endometrium, and placenta). We also discuss putative mechanisms of interaction between such microbial communities and various aspects of the immune system, with a focus on the characteristic immunological changes during normal pregnancy. Furthermore, we discuss how abnormal microbiota composition, “dysbiosis,” is linked to a spectrum of clinical disorders related to the female reproductive system and how the maternal immune system is involved. Finally, based on the data presented in this review, the future perspectives in diagnostic approaches, research directions and therapeutic opportunities are explored.

## Introduction

### Impact of Microbiome Research on Health Care

The human microbiome represents the collection of microbes living inside and on the surface of human body. Research efforts by the scientific community worldwide are increasingly focused on understanding the role of microbiota in health and disease. Since the completion of the Human Microbiome Project (HMP), and publication of the characteristics and functions of human microbiota located in different body habitats, this field of science has gained momentum (1). Current microbiome research tries to fill in the missing details in the pathophysiology and explain the seemingly random variation in disease severity and phenotype of confounding factors such as ethnicity, geographical location and societal habits. Due to advances in microbiome research, scientists have obtained valuable insight in many complex disorders such as obesity, cancer and inflammatory bowel disease, and a similar trend is observed in female reproductive tract in both physiological and pathological states (2).

### Global Burden of Pregnancy Complications

Because of the invasive nature of sampling methods, microorganisms populating the female reproductive tract remain less explored compared to microbiota populating the intestines; nonetheless, they represent an appreciable proportion (around 9%) of the female microbial network (3). Furthermore, disrupted female reproductive tract microbial communities have been implicated in reproductive and pregnancy complications, as reviewed in (4, 5). Reproductive and pregnancy complications are of global health interest, and comprise diverse health problems that occur prior to conception and during gestation. Notably, these involve the mother’s health, the baby’s health or both. Such problems may entail difficulties to conceive, or arise throughout gestation and span from the inability to maintain pregnancy in the first weeks of gestation, to its early termination in the third trimester. An increasing body of evidence associates microorganisms (including mutualistic) to the onset of reproductive health and maternal-fetal conditions, and to some of the major obstetrical syndromes, including premature birth, premature rupture of the membranes, premature labor, intrauterine growth restriction, and stillbirth (6).

The inability to conceive is an often neglected health concern affecting individuals around the globe. In 2010, an estimated 48.5 million couples worldwide were infertile, with little changes over the previous two decades (7). Early pregnancy loss (frequently before 13 weeks of gestation) is estimated to occur in 15–20% of recognized pregnancies, without major geographical differences (8–10). Of note, morbidity and pregnancy complications at more advanced stages of gestation (e.g., preeclampsia, preterm labor and stillbirth) often have a higher burden in low-resources settings, especially in south Asia and sub-Saharan Africa (11). Among such late complications, preterm birth remains the leading cause of worldwide neonatal morbidity and mortality: approximately 10.6% of all live births in 2014 were preterm, 80% of which occurred in Asian and sub-Saharan African countries (12).

### Current Gaps in Knowledge

Over recent years technological advancements, in particular sequencing-based methods for bacterial detection (metagenomics and 16S rRNA gene amplicon sequencing), have greatly expanded the literature on diverse microbiota colonizing the female reproductive tract both in healthy and disease states (5, 13). However, the wide spread application of sequencing-based methods incorporates a potential for false-positive results (i.e., low specificity) due to contamination as in case of the placenta (14). Our current knowledge is limited on how these microbiota interact with host cells, including local immune mediators, and whether this interaction is causally involved in the pathogenesis of pregnancy complications. It is well known that the mechanisms pivotal in regulating the establishment and maintenance of pregnancy, including epigenetic regulation and immune adaptation, are directly affected by local microorganisms (5, 13). Furthermore, the impact of maternal factors such as BMI, pre-existing disorders or life style habits (e.g., diet and nutrition) on this host-microbiota interaction need to be integrated and thoroughly studied in future studies (15). Understanding the interplay at the fetomaternal interface is essential for developing predictive human biomarkers for implantation and placentation and is a key step toward designing novel therapeutic approaches.

### Aims

This review aims to present an account of currently available literature on reproductive tract microbiota, describing the composition and function of microbiota and their links to pregnancy complications. The potential mechanisms of interaction between microbes and the immune system are discussed with respect to specific locations, focusing on the unique changes that characterize physiological pregnancy. Furthermore, the clinical impact of abnormal microbiota composition, i.e., “dysbiosis,” on female reproductive biology, from the pre-conceptional stage throughout early and late pregnancy. Common methodological challenges confronting research in the field related to study design and technical issues of sample collection and assay standardization are highlighted. Finally, we present options with respect to translation of various aspects and insights to future therapeutic approaches in reproductive medicine.

## Composition of Reproductive Tract Microbiome in Relation to Pregnancy

### Vaginal Microbiota

The community of microbes in the female lower genital tract plays a fundamental role in the promotion of homeostasis and in the prevention of colonization by pathogenic microorganisms. Compared to other sites, the vagina appears to harbor particularly simple microbial communities of low diversity (1). Although relatively simple at the genus-level, the diversity of *Lactobacilli* in the vaginal space is nonetheless higher than at other body sites (1). Many studies among non-pregnant women of reproductive age report that *Lactobacillus* spp. is the predominant species in the vagina, although the possibility of normal vaginal microbiota dominated by bacteria other than Lactobacilli seems to be plausible [as reviewed in (16)]. Current evidence suggests that the vaginal bacterial community is in a state of dynamic equilibrium (17). The composition of the vaginal microbiota correlates with the most dominant bacterial community composition (i.e., community state type; CST) across time (17). This notion appears to be in line with the concept of community resilience. Community resilience suggests that the ability of a microbial ecosystem to mitigate composition changes depends on the presence of beneficial species with stabilizing roles. For instance, *Lactobacillus crispatus*-dominated communities are less likely to transition to non-beneficial CST, than communities dominated by other *Lactobacillus* spp., like *L. iners* (17, 18).

In non-pregnant women of reproductive age, transient variations in the vaginal microbiota’s dynamic equilibrium are the results of physiological changes in response to hormones during the menstrual cycle, or human activities (e.g., sexual intercourse and hygienic practices) (18). In addition, other constitutional and environmental factors, including age, ethnicity, geographical variation and sexual habits influence the bacterial communities detected in the lower urogenital tract (19–21). For example, Lactobacilli-dominated vaginal microbiota has been shown to be less prevalent among non-Caucasian women in several studies (22–24), though, when present, their beneficial role seems maintained. A well-known study performed in North America characterized the vaginal microbiota of women of reproductive age from four ethnic groups (Caucasian, African-American, Hispanic and Asian): five groups of microbial communities, called Community State Types (CSTs) were identified. Whereas CST-IV was the most diverse CST and was also associated with a higher local pH, the remaining four (CST-I, CST-II, CST-III, and CST-V) were dominated by Lactobacilli (20). Lactobacilli thrive in anaerobic environments and produce lactic acid, therefore contributing to the acidic vaginal environment. Several studies have reported how depletion of these microorganisms often leads to vaginal dysbiosis, which is occasionally symptomatic, and at times associated with several important reproductive complications (25, 26).

The microbiota residing in the cervix have been sparsely studied as an independent entity. Current evidence suggests a strong similarity between bacterial communities in the cervix and in the vaginal area, suggesting ascending bacterial colonization from the vagina to the cervix (27). Such microbial communities are sometimes jointly referred to as cervicovaginal microbiota (28).

While in non-pregnant reproductive age women the vaginal microbiota is relatively dynamic, in healthy pregnancies it is characterized by an increase in stability (29). This is one of the physiological changes taking place in response to gestation. Other changes in this microbial community located in the vaginal area are an overall decrease in richness (number of different species present) and diversity (of the microbial ecosystem, i.e., the relative abundance of species). Overall, abundance of *Lactobacillus* spp. appears to be higher in healthy pregnant women, than in women with complicated pregnancies; whereas *Mycoplasma* and *Ureaplasma* appear to be lower (30). Most of the studies in the field seem to agree that Lactobacilli, in particular *L. crispatus*, *L. iners*, *L. jenseii*, and *L. gasseri* are the dominant bacteria detected in the vaginal microbiota of pregnant women (29, 31, 32).

Some of the factors influencing vaginal microbiota’s diversity during pregnancy are: gestational age (32), previous pregnancy history (33) and ethnicity (31, 34). In particular, *L. crispatu*s was the most dominant species in a Caucasian cohort (35), *L. iners* in an African American one (29) and, surprisingly, *L. acidophilus* in a mostly Hispanic population where *L. crispatus* was not detected, even though tested for (36). Although ethnic and geographical differences in vaginal microbiota are not yet understood, nor consistently observed (32), a combination of colonization by gut microbes, hygienic and sexual practices, and host genetics may contribute to underlying mechanisms (37).

As pregnancy progresses, the vaginal microbiota changes with an increase in the relative abundance of Lactobacilli and decrease in anaerobe or strict-anaerobe species, until around 36 weeks of gestation, when the number of species increases significantly (31). Such composition has been reported to resemble that of the vaginal microbiota in the non-pregnant state (34). Throughout pregnancy, some species of Lactobacilli associate with “normal” (i.e., healthy) vaginal microbiota, whereas the presence of other species reflect an abnormal, less beneficial microbial community. The former has been reported for *L. crispatus*, whereas the latter for *L. gasseri* and *L. iners* (35). Finally, reported changes in the vaginal microbiota after delivery include a decrease in *Lactobacillus* species and an increase in anaerobe ones (38), irrespective of the vaginal communities during pregnancy and independent of ethnicity (39).

It is becoming increasingly apparent from studies in the field that, besides investigation of the vaginal microbiota as an ecological community (defined by its most abundant species), individual (even low abundant) species with less beneficial roles should be considered, as their mere presence might be sufficiently detrimental to a healthy microbiotal equilibrium.

### Endometrial Microbiota

Unlike the vagina, the endometrium has not been extensively studied as a site of commensal bacterial colonization, in part likely due to the relatively limited access to uncontaminated samples. Historically, the uterus has been considered sterile (40), and presence of bacteria in endometrial, placental or amniotic fluid samples was viewed as pathological. However, with the advent of culture-independent techniques, recently compiled data support both the prevalence and variation of bacterial communities in the endometrium and their possible role in reproductive health (41) but is still under debate (14). Together, much of our knowledge comes from the interpretation and comparison of the role of microbiome in anatomically related sites.

In healthy non-pregnant women, the endometrium appears to harbor a unique, low-biomass microbiota, dominated by a few bacterial species including *Bacteroidetes* (*Flavobacterium* spp.) and *Firmicutes* (*Lactobacillus* spp.) (5, 41, 42). Compared to the vaginal microbiome, the endometrium harbors a significantly lower quantity of microbes, suggesting that the cervix and/or uterine environment serve as a barrier for ascending microorganisms (42). Since Lactobacilli and Streptococci represent the most dominant bacteria in the vagina and in the cervix, respectively, the occurrence of these species in the uterine cavity may indicate contamination during sample collection. Therefore, the use of paired samples from vagina, cervix and endometrial fluid has been proposed in order to facilitate interpretation (43).

A recent review reported that, in studies using culture-dependent techniques, no bacterial family was reported more than once in uterine or endometrial samples, indicating one of the challenges in determining the complexity of “normal” reference microbiota of the uterine cavity in this manner. However, in culture-independent analyses, *Lactobacilli*, *Bifidobacteriaceae*, *Comamonadaceae*, and *Streptococcaceae* were reported more than once in the uterine cavity, suggesting that the culture-independent technology approximate microbiotal complexity more truthfully (41).

### Placental Microbiota

The notion that the uterus represents a sterile environment during pregnancy was first challenged with the advent of the Human Microbiome Project (HMP) (1) and other studies (44). The detection of unique bacterial DNA profiles in human placenta supported the idea that a rich microbial community normally exists *in utero*. Surprisingly, these early data suggested that the placental microbiome resembles the oral cavity microbiome rather than that of adjacent sites, e.g., the vagina. Since these early reports, several reservations on this notion and its implications on pregnancy complications have been voiced, noting that the low-biomass that bacterial DNA in the placenta represents, is sensitive to capturing background contamination (from DNA extraction kits, polymerase chain reaction reagents, and laboratory environments) (45, 46). Recent meticulous analyses of metagenomic DNA, consistently found no significant differences in the abundance and/or presence of a microbiota between placental tissue (term women without labor) and background technical controls (14, 47). The proposed link between oral dysbiosis and pregnancy complications puts the debate about placental microbiota in the focus: clinical studies on the association between gingivitis and PTB have reported bacteria in the very old structures of the placenta (48). In the context of placental microbiome analysis, the pitfalls associated with technology of choice, in addition to the methodological problems, needs careful consideration (see section “Discussion,” diagnostic challenges).

## Maternal Immune Response in Pregnancy

During normal pregnancy, the immune system of the female reproductive tract is uniquely challenged by the fact that it has to protect against invading pathogens while simultaneously tolerating and supporting implantation and growth of the semi-allogenic fetus in a tightly regulated process (49). The implantation phase is characterized by low grade pro-inflammatory immune reactivity, including the production of major cytokines IL-6, IL-8, and tumor necrosis factor (TNF)-α. This response is believed to support local repair of endometrial injury and removal of cellular debris during trophoblast invasion and implantation. The placentation phase is predominantly an anti-inflammatory state, which is needed to ascertain tolerance of maternal immune cells to paternal antigens expressed by placental trophoblasts for the fetus and, at later stages, *vice versa*. The final parturition phase again requires controlled pro-inflammatory immune reactivation to trigger labor, delivery and placental rejection (50). Dysregulation of this tight immunological balance, based either in the (immuno)genetic constitution of the parents or the (local) uterine environment, may underlie several pregnancy complications. The homeostasis of the female reproductive tract during pregnancy depends on the interactive protective roles of epithelial defenses and immune cells. A broad variety of innate-(predominantly macrophages, dendritic cells and innate lymphoid cells) and adaptive immune cells (especially T cells) can be found each in different anatomic compartments with their own unique and specialized role. This section summarizes key immune players involved in immuno-modulatory events that support normal pregnancy, both the genetic and environmental aspects will be discussed in successive paragraphs. The maternal immune regulation in pregnancy has been extensively described (51) and is beyond the scope of this review.

### Epithelial Defenses

Like in many other mucosal tissues, the female reproductive tract’s first line of defense against pathogens is a physical barrier that, among other things, consists of a mucous layer, IgA antibodies and commensal bacteria limiting colonization by pathogenic bacteria. In addition, epithelial cells lining the female reproductive tract physically block pathogen invasion and produce protective molecules like antimicrobial peptides (AMPs). AMPs are multifunctional molecules with important roles in direct microbial killing, protection against proteolytic enzymes from various pathogens including bacteria, fungi, and some viruses, and modulation of both innate and adaptive immune responses (52, 53). AMPs are produced either constitutively or after induction by inflammatory stimuli, and are present on the mucosal surface, in decidual stroma, in endometrial fluid and even in amniotic fluid (54). In humans, AMPs have been linked to key regulatory processes in implantation and are implicated in the pathogenesis of various pregnancy complications (52, 55). An example of an AMP found in the uterus is the secretory leukocyte protease inhibitor (SLPI), which has antiviral and antifungal properties, and acts as a bactericidal against gram negative as well as gram-positive bacteria such as *Escherichia coli* and *Staphylococcus aureus* (56).

### Uterine Natural Killer Cells

Natural Killer (NK) cells are a critical component of the innate immune system and comprise up to 70% of all endometrial leukocytes during the secretory phase of the menstrual cycle and in early pregnancy, but decline in numbers by mid-gestation (49). The main function NK cells in the vagina is to provide protection against a broad variety of viruses. Like their blood counterparts, vaginal NK cells recognize virus- or stress-associated molecules and they destroy infected cells through the release of toxic granules containing granzymes and perforins or though interaction with death receptors (57). The exact function of uterine NK cells (uNK) is not completely clear as they clearly differ from NK cells in peripheral blood in surface markers and cytokine repertoire. uNK cells are poorly cytotoxic, but are a potent source of cytokines such as Interferon (IFN)γ, TNF-α, Granulocyte-macrophage colony-stimulating factor (GM-CSF) and Interleukin (IL)-10 as well as proangiogenic factors like vascular endothelial growth factor (VEGF) (58–60). Studies using genetic mouse models lacking uNK cells provided histological evidence of failed trophoblast invasion and defective spiral artery remodeling and highlighted the critical role for uNK in for normal placentation (61, 62).

### Macrophages and Dendritic Cells

Myeloid cells (macrophages and dendritic cells) represent the second most abundant immune cell subset in the endometrium and account for 10–20% of the decidual leukocyte population (49). They are responsible for the surveillance and scavenging of bacteria present on mucosal surfaces and act as antigen presenting cells (APCs) (58, 63, 64). APCs are equipped with pattern recognition receptors (PRR), such as Toll-like receptors (TLRs), allowing them to recognize the so-called pathogen-associated molecular patterns (PAMPs). PAMPs are species-specific and include molecules from the microbial cell wall (e.g., peptidoglycan) and cell membranes (e.g., LPS) or virus-derived single stranded DNA or double stranded RNA. PAMPs promote the production of cytokines and cell adhesion molecules that lead to the recruitment other immune cells such as neutrophils and NK cells. In addition, APCs have a major function in instructing the adaptive immune response by virtue of expressing major histocompatibility complex (MHC) class II receptors. In contrast to uNK cells, the numbers of decidual macrophages remain relatively constant throughout gestation, however, the diverse repertoire of macrophages in cytokines production, in regulation of T cell responses and in tissue repair suggest an important role in decidualization (49, 59). Together with the uNK cells, uterine macrophages are also postulated to facilitate angiogenesis during placentation and more importantly, spiral artery remodeling by production of growth factors and clearance of cell debris (60, 65).

Uterine dendritic cells (uDC) have a tolerogenic phenotype and both uDC and uterine macrophages produce IL-10, TGF-β, and indolomine2,3 (IDO) contributing to a tolerogenic and receptive micro-environment (66). In addition, uDC have been shown to interact in a bidirectional manner with uNK cells in mouse models, as well as *in vitro* human studies (67, 68). As a subclass of APC, uDC promote uNK differentiation and activation in a contact-dependent manner and via the production of IL-15 (69).

### T Lymphocytes

Adaptive immune cells (B- and T lymphocytes) provide highly specific and long-lasting cellular and humoral immunity against pathogens. While B cells are relatively infrequent in the female reproductive tract, T cells can be consistently found in both the vaginal compartment as well as in the uterus, albeit, in the uterus, their number and phenotype highly vary depending on stage of the menstrual cycle and pregnancy. CD8+ (cytotoxic) T cells represent the most abundant adaptive lymphocyte subset in the pregnant uterus (70). CD4+ (helper) T cells are less abundant, however, important in production of cytokines and interaction with other immune cells upon activation by APC (71). Traditionally, pregnancy was considered an immune privileged state by the different cytokines produced from a dominant T-cell phenotype (Th2) involved in immune tolerance, over another phenotype (Th1) involved in immune rejection. The dominance of Th1 polarized T cells was considered detrimental to embryo implantation and was associated with obstetric complications mainly preeclampsia (72). More recent studies show that the Th1/Th2 paradigm is not inclusive enough and that fetal tolerance is a complex process involving more specialized T cell subtypes, such as Th17 and regulatory T (Treg) cells (51). In humans, Treg cells have been shown to migrate from peripheral blood to the decidua (73), and their levels peak during the second trimester of pregnancy (74). They produce IL-10, leukemia inhibitory factor (LIF), transforming growth factor (TGF)-β, and heme oxygenase 1 (HO-1) contributing to fetal-maternal immune tolerance (75). It is clear that tight regulation of T cell activation and polarization is essential to balance the protection from pathogens, mediated by Th1/Th17 CD4+ T cells and CD8+ (cytotoxic) T cells *versus* tolerance to paternal antigens expressed by fetal cells, mediated by Th2 and Treg cells (76).

The induction of regulatory T cells (suppressor T cells/Treg) is favored over pro-inflammatory Th17 cells through interaction of uDC and uNK cells (77), corroborating the importance of intricate immunological instruction in acquisition of tolerance during implantation. Both macrophages and uDCs can be activated by encountering pathogens in the endometrium and start the process of phagocytosis, internalization, and degradation of the components of the antigen. They subsequently present these bacterial peptides to T-cells via MHC receptors, which activate the T-cells to initiate a cell-mediated and/or humoral immune response via MHC class II molecules (78).

Even though the intricate role of the immune system in pregnancy is not completely understood, collaborative action of innate- and adaptive immune cells appear to be critical for orchestration of the immunological changes required for successful fertilization, implantation and pregnancy.

## Interaction Between Maternal Immune System and Reproductive Tract Microbiota

**FIGURE 1:**
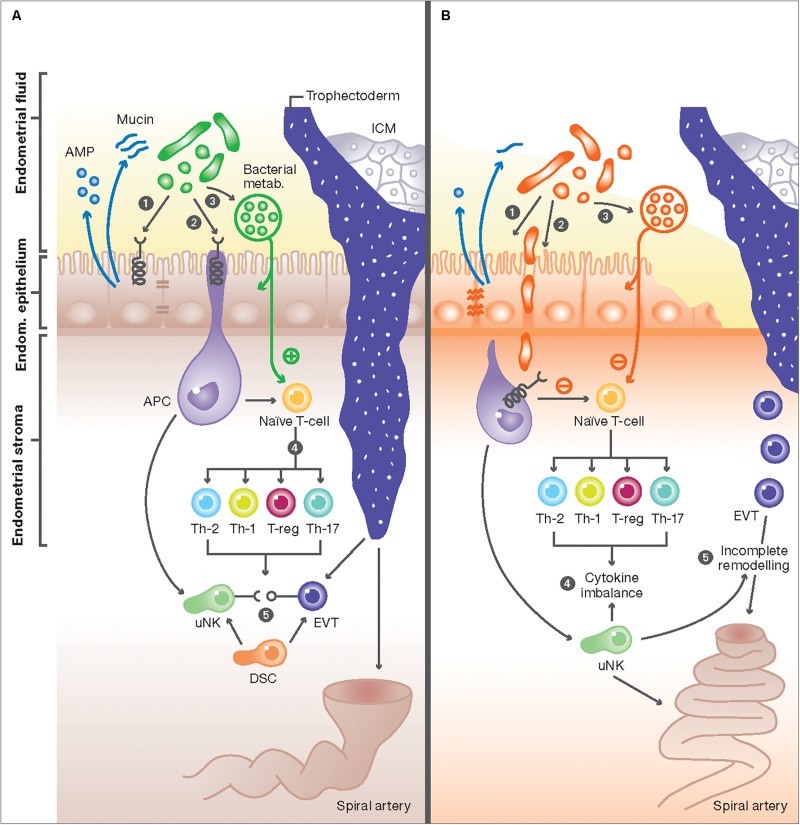
The interaction between the endometrial microbiome and local immune mediators. **(A)** In healthy women, commensal bacterial communities interact with immune cells with at the feto-maternal interface through three potential mechanisms: (1) Commensal bacteria (green) maintain a healthy physical barrier by stimulating the production of different antimicrobial peptides (AMP) from endometrial cells and preserving epithelial tight junctions and stable mucus production. (2) Once encountered by immune cells in the endometrium, e.g., antigen presenting cells (APC), commensal bacteria trigger signal transduction via pattern recognition receptors (PRR) through their pathogen-associated molecular patterns (PAMPs). (3) Commensal bacteria can also produce metabolites, such as polysaccharides and short-chain fatty acids (SCFAs), that potentially affect immune responses in endometrial epithelial cells and T-cells, or alter the endometrial fluid pH to produce a competitive niche microenvironment against pathogenic bacteria. These mechanisms result in activation of uterine NK (uNK) cells and the development of specific subsets of T-cells, characterized by high number of regulatory T-cells (Treg), low number of Th-17, and a switch from the Th1 to Th2 cytokine production (4). The interaction of activated uNK cells (KIR receptors) with HLA-C and -G from extravillous trophoblasts (EVT) from the implanting embryo will also promote EVT invasion, stromal matrix degradation, angiogenesis and ultimately the remodeling of maternal spiral arteries (5). These adaptive changes ensure an immunotolerant milieu for the semi-allogenic fetus and are essential steps essential step in normal placentation. **(B)** Disturbance of the normal endometrial microbiome can negatively impact the implantation process: (1) First, the dominance of non-commensal bacterial communities could weaken the integrity of the endometrial mucosal barrier by affecting the epithelial tight junctions and reducing AMP and mucin secretion. (2) This in turn will further weaken host defense mechanisms and allow pathogens to enter the endometrial stroma and elicit a profound immune reaction from APC and other immune cells harboring pattern recognition receptors (PRR). (3) Aberrant stimulation of T-cells, either directly from invading pathogens breaching the mucosal barrier or indirectly from absorbed bacterial products results in disbalance in cytokine production in favor of the pro-inflammatory Th-1 types, predominated by TNF-a, IFN, IL-2, and IL-10 (4). Aberration in uNK cell maturation, either primary or secondary to shallow EVT invasion, is a possible link between disturbed endometrial microbiome and incomplete remodeling of maternal spiral arteries, characteristic of the great obstetric syndromes (5).

The interaction between microbiota and the immune system is a complex process that is crucial for maintaining normal homeostasis in organs, albeit under the influence of several constitutional and environmental factors. We hypothesize that under normal circumstances, a healthy lifestyle (including diet, physical and psychological aspects) would result in a normal reproductive tract microbiota “eubiosis,” kept in check by a well-balanced immune regulation (Figure 1). Disturbance of this delicate balance could lead to either to inappropriate immune response and an exaggerated inflammatory reaction, or to downregulated immune response and dominance of pathogenic bacteria over normal commensals “dysbiosis”. Evidence on the existence of such balance during in pregnancy is limited and hence, the data on host-microbiota interaction discussed in this section are largely derived from the non-pregnant population.

### Host-Microbiota Interaction in the Vagina

#### Vaginal Protection by Lactobacilli

The vaginal mucosa is a barrier that provides protection against invading pathogens, as a result of the interaction between its epithelial cells, the immune system, and symbiotic microorganisms (79). The microbiota residing in the vaginal space are an active critical component in such defense system against infections. In particular, *Lactobacillus* spp. are thought to protect the upper genital tract from ascending infection, such as sexually transmitted ones (80). The main mechanism associated with the protective effect *Lactobacillus* spp. is the ability to produce lactic acid, thus maintaining a local pH of <4.5, deleterious to pathogens (81). Another defense mechanism is the Lactobacilli’s production of bacteriocins, which directly inhibit or kill bacterial and viral pathogens (81). The ability to form micro-colonies that adhere to epithelial cells and prevent adhesion of pathogens is additional means of defense by vaginal microbiota, as is their ability to trigger the host’s defense (81). *In vitro* studies have shown that certain *Lactobacillus* species are able to temper inflammation by, for example, a reduction of IL-6, IL-8, and TNF-α secretion after bacterial stimulation of toll-like receptors (TLRs) (82). The association between Lactobacilli-poor vaginal ecosystems and an increased risk of sexually transmitted infection is strong [as reviewed in (80, 83)]. Nonetheless, non-Lactobacilli*-*dominated vaginal microbiota occur in 25% of asymptomatic women, which challenges the notion of Lactobacilli as the sole microbial defense mediator (17, 84). Possible other explanations may be that maintenance of low pH in non-Lactobacilli*-*dominated vaginal microbiota is achieved in a different manner (18), or that not all suboptimal microbiota are manifested as symptomatic, whereas such CSTs may nevertheless correlate with increased risk for adverse reproductive health outcomes (85). Furthermore, as discussed before, distinct *Lactobacillus* species appear to differentially affect microbiota, e.g., with *L. iners* being more conductive to pathogen invasion than *L. crispatus* (25).

#### Hormonal Regulation of Host-Vaginal Microbiota Interactions

Most evidence and knowledge on the interactions between vaginal microbiota and host immune system comes from studies in non-pregnant women and should be extrapolated with caution to infer host-vaginal microbiota interactions in pregnancy. Key regulators of this interaction are sex hormones, which regulate the release of pro-inflammatory cytokines, chemokines and antimicrobial peptides, and contribute to the selection of vaginal microbial species [as reviewed in (86)]. In particular, estradiol has been implicated in the shift from a *Lactobacillus*-poor to a *Lactobacillus*-rich vaginal microbiota during puberty, as well as a reverse shift after menopause (86). Estrogen-induced glycogen synthesis in epithelial cells and production of glycogen-metabolites (maltose, maltotriose, α-dextrines) provides substrates for conversion to lactic acid by Lactobacilli (87–89). At reproductive age, a healthy vaginal microbiota was found to amplify the fluctuation in local immune responses in synchrony with hormonal changes during the menstrual cycle (90). In particular, women with a *Lactobacillus*-poor vaginal microbiota altered hormone-associated immune change may correlate with an increase susceptibility to infections (90). The close interplay between immune status and vaginal microbiota composition is further supported by the correlation of a less beneficial vaginal immune signature with ongoing HIV infection status in post-menopausal women (91).

#### Vaginal Immuno-Microbiotal Interactions

As an integral part of the defense mechanisms of the vaginal space, the local microbiota is required to interact with the host immune system. The ability of the host to protect against pathogenic microorganisms, but not react against the symbiotic microbes residing in the vagina, relies on the bi-directional relationship between immune system and microbiota (92). Such interplay helps maintain an immune-tolerant environment, more so during pregnancy. As a result of this symbiotic tolerance, bacterial communities thrive in the vaginal environment, contribute to local immune defense. Conversely, dysbiosis of the vaginal communities has been implicated in the disruption of the mucosal layer, decreasing the ability of the mucus and vaginal secretions to trap and inactivate pathogens. This might also facilitate the formation of epithelial entry portals for the same pathogens (93).

A possible mechanism for vaginal dysbiosis is the increased production of pro-inflammatory cytokines and chemokines, associated with the increase in pathogenic microbial diversity, which contributes to further recruitment of immune cells and amplification of the inflammatory response [reviewed in (86)]. Clinical studies performed on vaginal samples from sub-Saharan Africa, have shown a correlation between the presence of a non-*Lactobacillus* dominant microbiota and a rise in inflammatory cytokines and chemokines in the vagina (94, 95). Selected non-beneficial bacteria found in the vaginal tract induce pro-inflammatory cytokines and chemokines in *in vitro* co-cultures with vaginal epithelial cells. Relevantly, *L. crispatus*, the most well-known beneficial vaginal microorganism, does not induce inflammatory cytokine release in such settings [reviewed in (82, 96)]. In accordance with this, another study had shown how the possible contribution of vaginal dysbiosis to infections of the urinary tract was mediated by defects of the immune response (97).

Healthy vaginal microbiota was associated, both *in vitro* and *in vivo*, with increased expression (mRNA and protein) of defensins, specific types of vaginal antimicrobial peptides (AMP) that prevent binding of pathogen-specific proteins to human cells. AMP levels were significantly lower in bacterial vaginosis conditions, *in vitro* and *in vivo* (98). The expression of other types of antimicrobial peptides, the secretory leukocyte protease inhibitor and the human epididymis protein 4, correlates with the presence of less beneficial vaginal microbes (96). The complement system was proposed as a key player in adverse pregnancy outcome, as female microbiota composition regulates complement function in the maternal vasculature (99). Complement dysregulation in the intrauterine space, promotes inflammation and triggers a cascade of physiological changes (cervical changes, degradation of collagen, uterine decidua activation and uterine contractility), which in turn increases risk for preterm delivery (97).

Such combined evidence suggests that the vaginal microbiota modulates the local immune system and inflammatory response at least in part through interaction with epithelial cells, thereby influencing the susceptibility to infection. Although interactions between the maternal immune system and vaginal microbiota appear to be complex and far from completely understood, based on the above outlined observations it was suggested that the overall microbiota composition, rather than any individual microbial population, underlies adverse interactions with the host-immune system in the female reproductive system (100).

### Host-Microbiota Interaction During Implantation and Placentation

Successful implantation and subsequent formation of the placenta (placentation) encompass several steps ensuring tissue adhesion between fetal trophoblasts and maternal tissues and the adaptation of their blood vessels and to facilitate nutrient supply (101). These steps involve mechanisms such as angiogenesis (101), decidualization (102) and immune response adaptation (60). This immune adaptation is essential in pregnancy and is required in order to avoid a graft *vs.* host disease between the semi-allogenic trophoblast and maternal tissues, including immune cells, decidual microbiota and other decidual components such as epithelial and stromal cells and blood vessels. Not surprisingly, this complex interaction is postulated to affect subsequent stages of pregnancy, and is implicated in many pregnancy complications (103).

Published literature points to the existence of a diverse and metabolically active endometrial microbiota and predicts an important physiologically modulatory role of the main function of its host tissue: harboring and nurturing the developing embryo. In healthy women, the presence of commensal bacterial communities in the cycling endometrium mediate physiological responses from various cells at the feto-maternal interface, including epithelial and stromal cells in the endometrium, immune cells and trophoblasts from the implanting embryo. Although the exact molecular nature and extent of these interactions are not fully understood, evidence from *in vitro* experiments and animal models have provided important insights (13, 55, 104, 105). Based on currently available knowledge, we postulate the following mechanisms:

1) Commensal bacteria interact with endometrial epithelial cells to maintain a healthy physical and antimicrobial barrier against pathogens. The binding of commensal bacteria to epithelial cells triggers the release of various antimicrobial peptides (AMPs) into the uterine cavity, which constitute part of key defense mechanisms of epithelial tissues against a proteolytic enzymes from various pathogens including bacteria, fungi, and some viruses (52, 53). In addition to the production of AMPs, commensal bacteria induce a biochemically neutral and biophysically stable mucus production by endometrial cells and stabilize the adherens junctions and tight junctions (55, 106). The maintenance of an intact and stable epithelial barrier is an integral part of the natural defense strategies in preventing the colonization and penetration protecting the endometrium from opportunistic microbial infections.
2) Commensal bacteria can alter the immune response at the cellular level through numerous components of the innate and adaptive immune system in the endometrium. The key sensors of bacterial presence in tissues are antigen presenting cells (APCs), in the endometrium they are represented by macrophages and dendritic cells (uDCs) (see section “Maternal Immune Response in Pregnancy”). Both cell types play an important role in maintaining tolerance against the commensal microbiota by modulating the immune response of other components of the innate and adaptive systems (63, 107). Macrophage-derived IL-10 is critical for Foxp3 + Treg cell development, maintenance, and expansion (77). uDCs also are a major source of IL-23 which, in combination with other cytokines, influences the differentiation of Th17 (77). Although both macrophages and DCs can process and present bacterial antigens, differences in their physiology and function suggest they have complementary roles in the immune response against bacteria.
3) The downstream effects of triggering the immune system at the feto-maternal interface by bacteria is the activation of uterine NK (uNK) cells and the development of specific subsets of T-cells, characterized by high number of regulatory T (Treg) cells, low number of Th-17, and a switch from the Th1 to Th2 cytokine production. These adaptive changes ensure an immunotolerant milieu for the semi-allogenic fetus and are essential steps in normal placentation. The interaction of activated uNK cells via specific receptors (KIR) with HLA-C and -G from extravillous trophoblasts (EVT) from the implanting embryo will also promote EVT invasion, stromal matrix degradation, angiogenesis and ultimately the remodeling of maternal spiral arteries.

How the host-microbiota interactions affect the maternal immune response during implantation is not clearly understood. One of the factors, which govern immune modulation and maternal tolerance is the Pre-Implantation Factor (PIF) (108). PIF is a 9–15 amino acid peptide secreted by viable placenta with high concentrations in the maternal circulation in the first trimester (108, 109). PIF shows local effects on the endometrium and trophoblast promoting implantation and invasion of the trophoblast and has a direct impact on immune cell function and targets neutrophils and macrophages, as well as CD4+ and CD8+ T-cells (109). PIF reduces the activation of the NALP3 inflammasome complex (mainly TLR-4 mediated) resulting in reduction of pro-inflammatory cytokines such as IL-1β, IL-18, and IL-33 (110, 111). In addition, PIF creates an anti-inflammatory milieu by reducing IFNγ and stimulating IL-10 secretion, as well as enhancing Th2 cytokines. In context of the macrophages responding to bacterial stimuli, PIF blocks the release of nitric oxide induced by lipopolysaccharides (LPS) (108). This effect is present in case of excessive stimulus only. Thus, PIF operates as an immune modulator, rather than an immune suppressor with minimal impact on the innate, but firm effect on the adaptive immune response (108). Additional protective effects on the embryo protection and development include targeting the protein-disulfide isomerase (PDI) and heat shock proteins (HSP), which impact oxidative stress and protein misfolding (112, 113). Overall PIF is an example of embryonal factor shaping maternal immune response and therefore promoting successful implantation by generating an anti-inflammatory milieu and facilitates immune tolerance. The interaction of microbiome and PIF on pregnancy complications are currently under investigation.

## Clinical Implications of Dysbiosis and Disturbed Immune System

**FIGURE 2:**
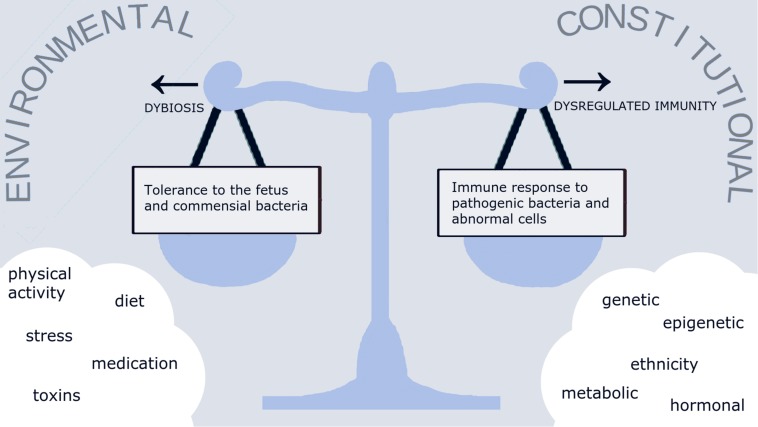
The conceptual relationship of reproductive tract microbiome and immune response. A multitude of environmental and constitutional factors affect the balance between tolerance to the fetus and commensal bacteria on one hand, and the immune response to pathogens and abnormal cells on the other hand. Loss of balance between the microbiome and immune responses would lead to either abnormal commensal microbiome and pathogenic infection “dysbiosis” or excessive immune reaction and sterile inflammation “dysregulated immunity.”

Abnormal composition and/or function of the of reproductive tract dysbiosis is implicated in various gynecological disorders and pregnancy complications (4, 114, 115) (Figure 2). Although many gynecological disorders have been linked to dysbiosis and can indirectly affect reproductive outcomes [e.g., endometriosis and ectopic pregnancy (55)], discussion of these specific complications is outside the scope of this review. Depending on the anatomical site within the female reproductive tract, dysbiosis is associated with various clinical disorders The clinical implications of dysbiosis of the female reproductive tract in relation to pregnancy ranging from infections: BV, bacterial vaginosis; PID, pelvic inflammatory disease; STI’s, sexually transmitted infections; early pregnancy complications: RM, recurrent miscarriage; RIF, recurrent implantation failure; and late pregnancy complications: pPROM, premature pre-labor rupture of membranes and placental dysfunction.

### Preconceptional Period (Genital Tract Infections)

As outlined in preceding sections, the immune system and microbiota play an interactive and collaborative role in maintaining a physiological healthy state in the reproductive tract. Disturbance of this physiological interaction has been implicated in the onset of diverse complications related to female reproductive health.

One of the largest longitudinal cohort studies that assessed vaginal microbiota vs. risk of STI is the Longitudinal Study of Vaginal Flora (LSVF) based in the United States (116). Women diagnosed with BV (by Nugent score assessment) had a nearly 2-fold increased risk of STI, like trichomonal, gonococcal, and/or chlamydial infection (of note: microbiota-testing preceded detection of STI by 3 months). This evidence is in agreement with previous reports that define vaginal dysbiosis as a predictor for gonorrhea and chlamydial infection (117). More recently, individuals with chlamydial infection were found more likely to have a cervicovaginal microbiota dominated by *L. iners*, or non-*L. crispatus* anaerobic bacteria (118), although not all associations were statistically significant and may depend on ethnicity (119, 120).

Human Papilloma Virus (HPV) and the Human Immunodeficiency Virus (HIV) represent sexually transmitted viral infections increasingly studied in association with reproductive tract microbiota. Women with detected or persistent HPV infections showed a more diverse vaginal microbiota, in studies conducted among African/Caribbean and Italian women and suggested an association with *Atopobium* spp. and *G. vaginalis* (121, 122). Among Nigerian women, the prevalent high-risk HPV (hrHPV) infection was associated with a decrease in Lactobacilli and abundance of anaerobes, particularly of the general *Prevotella* and *Leptotrichia* (123). In Asia an association between increased vaginal bacterial diversity and presence of HPV were reported, with a Korean study suggesting Fusobacteria, in particular *Sneathia* spp. to be particularly implicated (124). A Chinese study identified several microbial genera in hrHPV-infected women (*Bifidobacterium*, *Bacillus*, *Megasphaera*, *Sneathia*, *Prevotella*, *Gardnerella*, *Fastidiosipila*, and *Dialister*), while another set (*Bifidobacterium*, *Megasphaera*, *Bacillus*, *Acidovorax*, *Oceanobacillus*, and *Lactococcus*) in hrHPV-infected pregnant women. In pregnancy, this study showed an association between a more diverse cervical microbiota and HPV (125). In addition, several genera and species were associated to HPV positivity (*Ureaplasma parvum*), HPV negativity (*Brochothrix*, *Diplorickettsia*, *Ezakiella*, *Faecalibacterium*, and *Fusobacterium*), likelihood of reinfection (*Actinomyces*) or persistence (*Prevotella*, *Dialister*, and *Lachnospiraceae*) (126). Recent data also suggested altered microbiota in placenta, cervix and mouth in the presence of HPV infection (119).

Two decades ago, the absence of Lactobacilli was already associated with an increased risk of acquiring HIV infection in a cohort of Kenyan women (120). Similarly, vaginal dysbiosis was suggested as a contributor to the acquisition of HIV in Ugandan and Zimbabwean women (127). Conversely, Rwandan women with a *Lactobacillus*-dominated (particularly *L. crispatus*) cervicovaginal microbiota were less likely to be infected with HIV, hrHPV, as well as Herpes Simplex Virus 2 (HRV 2) (128). Low pH, due to lactic acid production by Lactobacilli, was suggested as a main strategy to prevent HIV infection, either by means of inactivating the virus, or inactivating T lymphocytes, thus decreasing their susceptibility to HIV infection (129). Other possible defense mechanisms of vaginal microbiota to HIV include the production of peroxide or bacteriocins by Lactobacilli, although their effect on viral biology is not fully understood (129). These findings are seemingly congruous with the protective role of Lactobacilli in urinary tract infections [as reviewed in (130)].

The above mentioned sexually transmitted infections, among others, have been extensively implicated in diverse reproductive tract complications, from infertility, to adverse pregnancy outcomes. A growing body of evidence collectively supports the association between vaginal dysbiosis and different genital tract infections and corroborates an important physiological role for microbiota in protection from, or susceptibility to pathogenic infections. Interaction and cross-regulation between the vaginal microbiota and the immune responses in the lower genital tract are therefore crucial in creating a protective environment against external pathogens, thus ensuring the right condition for the initiation of pregnancy.

### Early Pregnancy (Infertility and Recurrent Miscarriage)

In recent years, the availability of culture-independent sequence techniques has led to a rise in the number of studies investigating the association of disturbed vaginal and endometrial microbiome composition and reproductive failure, focusing mainly on implantation failure. In women undergoing *in vitro* fertilization (IVF), the percentage of vaginal and endometrial Lactobacilli were significantly lower than non-IVF patients and healthy volunteers (131). In addition, studies have shown that presence of various bacterial contaminants, such as Enterobacteriaceae, Streptococcus, Staphylococcus, and Gram-negative bacteria, from catheter tips at the time of embryo transfer had a negative impact on pregnancy outcome, as reviewed in (13). Using 16S ribosomal RNA sequencing of paired endometrial and vaginal samples from 13 fertile women and 35 infertile patients undergoing IVF, Moreno et al. showed that the presence of a non-*Lactobacillus*-dominated microbiota (defined as <90% *Lactobacillus* spp.) was associated with significant decreases in implantation (60% vs. 23%), pregnancy (70% vs. 33%), ongoing pregnancy (59% vs. 13%) and live birth (59% vs. 7%) rates (43). Using a similar approach in a cohort of 31 women, Bernabeu et al. showed that women achieving pregnancy after IVF (cryotransfer of a single embryo) showed a greater presence of *Lactobacillus* spp., while a trend toward higher alpha diversity in vaginal samples was found in patients who did not achieve pregnancy and no difference in beta diversity (132). In a recent large prospective cohort study, Koedooder et al., used a new technique “IS-PRO” (see section “Diagnostic Challenges”) to examine microbial profiles of vaginal microbiota in 192 women undergoing IVF. Women with a low percentage of Lactobacillus in their vaginal sample were less likely to have a successful embryo implantation (133). This failure was correctly predicted in 32 out of 34 women based on the vaginal microbiota composition, resulting in a predictive accuracy of 94% (sensitivity, 26%; specificity, 97%). Additionally, the degree of dominance of *Lactobacillus crispatus* was an important factor in predicting pregnancy: none of the women who had a negative prediction (low chance of pregnancy) became pregnant. Taken together, these data suggest that a balanced, less diverse vaginal microbiota, dominated by *Lactobacillus* species increased the chances of a successful outcome.

In women with recurrent reproductive failure, ascribing a causative or correlative connection to aberrant microbiota is controversial. This group is composed of women with recurrent miscarriage (RM) (defined as loss of two or more clinically or biochemically established pregnancies) (134) and women with recurrent implantation failure (RIF), defined as loss of two or more pregnancy losses after transfer of good-quality embryos (135). RM and RIF both have heterogeneous etiology, with diverse risk factors being implicated covering genetic, metabolic, hormonal, immune maternal aspects. Although several groups have studied microbiota disturbances in women with recurrent reproductive failure, the complex pathogenesis has hampered any meaningful conclusion on the role of reproductive tract dysbiosis in this early pregnancy disorder. Analysis of endometrial samples from women investigated for recurrent reproductive failure showed that the uterine microbiota was dominated by *Bacteroides* species in >90% of the women (35). However, dissimilarities in dominance of *Prevotella* spp. or *L. crispatus* due to possible contamination from the vagina limits the interpretation of these data.

Chronic endometritis (CE) is an inflammatory condition typified by dysregulated interactions between endometrial pathogens and the endometrium. Chronic endometritis is a persistent inflammation of the endometrium, characterized by the presence of plasma cells syndecan-1 (CD138) on immunohistochemical staining of endometrial biopsies [reviewed in (136)]. Although various pathogens have been implicated in causing CE, the most commonly reported species were common bacteria (*Escherichia coli, Enterococcus faecalis*, and *Streptococcus agalactiae*) in 77.5%, followed by *Mycoplasma*/*Ureaplasma* (25%) and *Chlamydia* (13%) (137).

Recently, research has focused on the role of CE in reproductive failure, with various studies reporting a wide range prevalence depending on the clinical characteristics of the studied group and reflecting heterogeneity of diagnostic methodology and definitions. Studies have found an increased prevalence of CE in women with recurrent pregnancy loss (13%) (138) and RIF (30%) (139), while the rate of CE in the general infertility population was suggested to be much lower, with a prevalence of 2.8% among 606 infertility patients (140). A recent meta-analysis of five studies (total of 796 patients) concluded that women receiving antibiotic therapy for CE did not show any reproductive advantage in comparison with untreated controls (141). However, patients with cured CE, confirmed by a repeat biopsy, showed higher clinical pregnancy rate (OR 4), ongoing pregnancy rate/live birth rate (OR 6.8) and implantation rate (OR 3.2) (141). The exact mechanism of how CE affects implantation is yet unknown, but a negative effect on endometrial receptivity by abnormal infiltration of plasma cells (B lymphocytes) and antibody production is suggested (142). Similarly, as the causal connection between CE and dysbiosis is unknown, it is unclear whether a status of dysregulated immune response in the endometrium is responsible for the higher prevalence of dysbiosis, or whether dysbiosis is the cause of CE.

**FIGURE 3:**
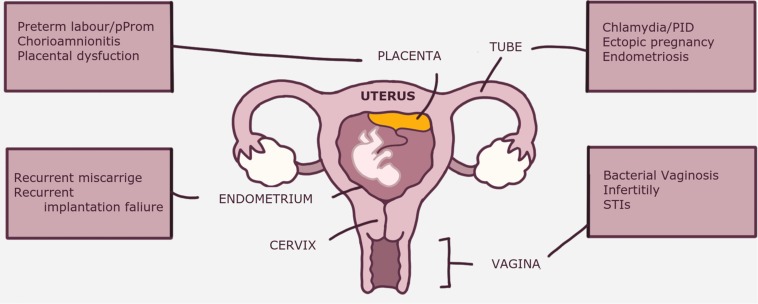
The clinical implications of dysbiosis (abnormal microbiota) of the female reproductive tract in relation to pregnancy. Some disorders, e.g., endometriosis are indirectly related to pregnancy disorders through their negative impact on fertility. BV, bacterial vaginosis; PID, pelvic inflammatory disease; PPROM, premature prelabor rupture of membranes; RM, recurrent miscarriage; RIF, recurrent implantation failure; STI’s, sexually transmitted infections.

How the disturbance of the endometrial microbial ecosystem can have a negative impact implantation process is not fully understood. Parallel to the proposed mechanism of interaction between the microbiota and endometrial cells, a disturbed balance can act upon the following mechanisms (Figure 3). (1) First, the dominance of non-commensal bacterial communities could weaken the integrity of the endometrial mucosal barrier by affecting the epithelial tight junctions and reducing AMP and mucin secretion; (2) This in turn could further weaken host defense mechanisms and allow pathogens to enter the endometrial stroma and elicit a profound immune reaction from APC and other immune cells harboring pattern recognition receptors (PRR); (3) Aberrant stimulation of T-cells, either directly from invading pathogens breaching the mucosal barrier, or indirectly from absorbed bacterial products, results in disbalance in cytokine production in favor of the pro-inflammatory Th-1 types, predominated by TNF-a, IFN, IL-2, and IL-10. Abnormal uNK cell maturation, either primary or secondary to shallow extravillous trophoblast (EVT) invasion, is a possible link between a disturbed endometrial microbiome and incomplete remodeling of maternal spiral arteries, characteristic of the great obstetric syndromes (143).

### Late Pregnancy (Premature Delivery, Premature Rupture of Membranes, Chorioamnionitis and Placental Dysfunction)

Alteration of the ecology of the female reproductive tract has been linked to maternal and fetal health, and to adverse pregnancy outcomes (27, 79, 144, 145). The most widely studied pregnancy complication in relation to the vaginal microbiota is preterm birth (PTB) (146–148). The prevalence of a *Lactobacillus*-poor microbiota was inversely correlated with gestational age at delivery in some studies (38), not in others (149). Distinctive species of Lactobacilli were reported to be associated with differential pregnancy outcomes. Indeed, in two ethnically distinct cohorts, *L. crispatus* was associated with a low PTB risk, whereas *L. iners* was not (42). Also, *Gardnerella* was also associated with PTB and coexisted with *L. iners*, but not with *L. crispatus* (42). Based on these findings, a model was suggested to help describe the interplay between key vaginal bacterial species: the presence of *G. vaginalis* correlates with adverse outcomes, such as PTB and symptomatic Bacterial Vaginosis (BV); *G. vaginalis* and *L. crispatus* are strongly mutually exclusive, while this is not the case for *L. iners* and *Gardnerella* (150). Comparison of vaginal samples of 90 women who delivered at term and 45 women with preterm birth suggested that a specific signature: the presence of BV-associated bacterium (BVAB) 1, *Prevotella* cluster 2, *Sneathia amnii* and BVAB-TM7 in early pregnancy, may be useful for prediction of PTB risk, particularly in high-risk populations of African ancestry (147).

Abnormal vaginal colonization in the second trimester was also associated with an increased PTB risk (151). Similarly, in a predominantly African-American population, an increased vaginal microbial community richness and diversity between the first and second trimester was associated with PTB (152). In addition to decreased *Lactobacillus* spp., specific pathogens have been linked to PTB in different populations, such as *Klebsiella pneumonia*, *Gardnerella, Ureaplasma* and other genera, including *Prevotella, Atopobium*, *Sneathia*, *Gemella*, *Megasphaera*, *Dorea*, *Streptococcus*, and *Escherichia/Shigella* (38).

At this point, although one out of four preterm births appears to be associated with intra-amniotic infection, and some associations between microbial states or abundance of individual species with PTB have been reported, it does not appear clear whether changes in the bacterial communities of the lower genital tract allow for a clear identification of women at risk (144). A recent overview summarizing studies on the association between vaginal microbiota and PTB emphasized the methodological heterogeneity, and scarcity of, studies in the field (146). Overall, research on the topic has produced conflicting outcome, often related to ethnical background of the women included and the associated risk degree of PTB. Nonetheless, more recent studies more consistently report an association between vaginal dysbiosis and PTB, possibly resulting from the improved understanding of the contribution of *L. iners* to this association (146).

Multiple studies provide evidence of placental and amniotic fluid microorganisms affecting miscarriage, chorioamnionitis, premature rupture of membranes (PROM), stillbirth, preeclampsia (PE), and intra uterine growth restriction (IUGR) rates (143, 153). In some cases of spontaneous preterm delivery, microorganisms have been found to invade the amniotic cavity, leading to increased maternal/neonatal morbidity and mortality (154). Infectious bacteria gain access, typically via ascending route and/or perturbations of the vaginal microbiota and can have an adverse impact on pregnancy outcomes (146). Chorioamnionitis is an inflammatory disease of the extraembryonic membranes, placenta and amniotic fluid due to microbial invasion mostly commonly *Ureaplasma* and *Mycoplasma* spp. infections (155). Culture-dependent studies identified members of the genera *Prevotella*, *Bacteroides*, *Peptostreptococcus*, *Gardnerella*, *Mobiluncus* and genital mycoplasmas in the placentae of women delivering preterm with or without PE, suggestion the involvement of multiple bacterial strains (15). In line with this, antibiotic treatments have not reduced the rates of preterm birth, suggesting that a single inflammatory/infectious pathway may not fully explain the problem (144). In contrast, DNA-based investigations of the placental microbiota in PTB showed increased enrichment of *Burkholderia* spp. and an increased relative abundance of Alphaprotoebacteria and Actinomycetales and mixed non-cultivable anaerobes (15). However, in case of chorioamnionitis a higher abundance of *Streptococcus agalactiae*, *Fusobacterium nucleatum* and *Ureaplasma parvum* was reported (15). These results fuel the debate whether a prenatal bacterial microbiota really exist (45). In physiological pregnancies or in the presence and absence of active labor, many “causal” microorganisms or their DNA are detectable in the placenta and amniotic fluid (156, 157). Since microbiotal ratios of specific microbial species change throughout gestation, the placental response to such environmental cues possibly does as well (158). This is supported by the observation that PIF is differently expressed in the placenta throughout gestation or in response to an inflammatory insult (109). Since, as of yet, the amniotic fluid and the membranes are not available for non-invasive sampling, the time of onset of chorioamnionitis is not available for clinical stratification and decision making. Besides placental inflammation, systemic inflammation in concert with oxidative stress and endothelial dysfunction plays a role in pregnancy complications such as IUGR or PE (159).

The interaction of vaginal and endometrial microbiota with local maternal components modulates the maternal immune system. Although the contribution of the fetal immune system to this interplay is currently unknown, it is conceivable that when the fetus initiates an inflammatory response, premature labor may impose a risk to fetal wellbeing. Conversely, there is an inherent risk that the fetus may be injured by a longer stay *in utero* either directly through microbial toxins or through proinflammatory cytokines. This delicate balance between maternal and fetal needs possibly dictates the course and outcome of pregnancy and may contribute to long-term health of the offspring. This may be another example how future health is primed by the intrauterine environment according to the principles of the DOHAD (Developmental Origins of Health and Disease) hypothesis (160).

## Future Perspective

### Diagnostic Challenges

As discussed throughout this review, one of the major limitations in microbiome research is the diversity of the techniques used and the databases linked to those techniques to identify individual species and determine the composition of microbiota. Such differences potentially introduce significant variations in analysis and constitute a major source of difference in interpretation of data. Although currently no compelling evidence points to the existence of a universal mammalian placental or fetal microbiota, consensus on the importance of uniform technological and analytical approaches warrants further investigation into standardization of microbiota research and incorporating geographical, ethnic and societal data (45) (Table 1). These recommendations are general for all microbial community profiles, including the female reproductive tract microbiota, and are expected to reduce variation and inconsistency between studies on microbiomes.

**TABLE 1:** Steps to obtain reliable microbiota*.

1. Larger sample sizes
2. Simultaneous use of different detection methods
3. The elimination of extracellular DNA prior to molecular microbial profiling
4. rigorous controls for reagents and equipment at all steps during sample processing and analysis
5. The determination of the relative abundance of bacterial groups should be preceded by an absolute quantification of the bacterial load in samples and controls
6. Prespecified and quantified bacterial mock communities to the examined samples will help to reveal biases and identify batch effects
7. Demonstration of metabolically active and proliferating diverse bacteria within the placental or fetal tissue will be required to prove the existence of a viable, diverse and unique bacterial community that merits the term microbiota.
8. Taking into consideration geographical, ethnic and societal habits of the population

*This table is adapted from the text and advices provided in Hornef M, Penders J. Does a prenatal bacterial microbiota exist? Mucosal Immunol. 2017 May;10(3):598–601.

Moreover, most of the techniques described have not undergone rigorous validation steps and certification by regulatory organs, such as the European commission *in vitro* diagnostic (CE-IVD) label or the American Food and Drug Administration (FDA) approval, limiting their commercial availability. The availability of CE-IVD-certified microbiome analysis tools would meet part of the recommendations (see: Table 1) and allow more reliable comparisons between studies in different countries and settings. Besides the widespread Next-Generation Sequencing (NGS) approaches, our group developed another detection technique called *IS-pro*, first described in 2009 (161). The *IS-pro* test is already CE-IVD certified and is currently being used in many different disease profiling activities and applications including analyses of the vaginal microbiome as a predictor for outcome of *in vitro* fertilization (133). A wider application of this technique to study the role of reproductive tract microbiome in various pregnancy complications is expected to be available in the near future.

### Fundamental Research Developments and Directions

The general concept of interactions between commensal microbiota and human cells has recently been embraced as a principal of human physiology. The realization that a dysbalanced and/or harmful microbiotal composition frequently correlates with specific clinical conditions, has led to a sharp rise in studies on host-microbiota interaction over the last decade (25, 55, 114, 162, 163). Although it is becoming increasingly clear that the interplay between host and microbiota also affects human reproductive biology, the exact molecular mechanisms underpinning these interactions are far from understood.

All long-lasting physiological adaptations of cells and tissues in response to altered environmental conditions have their basis in altered epigenomic programming. Environmental changes are detected by a myriad of cellular sensing mechanisms and, via signal transduction routes, ultimately reach the nucleus where environmental cues are translated to epigenetic regulation and chromatin remodeling. This epigenetic regulation of genetic input and potential environmental cues underlies maternal physiological plasticity and embryonal development during gestation. Programming of immune cells during immunological responses is mediated by cellular stress responses and is typically accompanied by metabolic changes in the cell. Prolonged disturbance of these interactions is associated with the development of immunological disorders, such as chronic inflammatory conditions (164). The close link between cellular metabolism and epigenetic responses has its origin in early evolution, has enabled multicellularity and is closely connected to organismal survival (165). Hence, fine-tuning of epigenetic control occurs in conjunction with cellular metabolic status, as available energy ultimately directs and limits cell responses. Recent advances in this field have identified oxygen, numerous metabolic intermediates, cellular reduction (NADH, FADH_2_) and energy equivalents (ATP, GTP), as direct molecular effectors of epigenetic regulatory activity (165). Conversely, cellular metabolic adaptation is controlled by epigenetic regulation, substantiating the reciprocal nature of the physiological interaction between metabolism and epigenetics (166). Sex hormones, metabolic profiles, nutrition, maternal stress, drugs, smoking and air pollution represent obvious examples of environmental cues that, via active modulation of epigenetic regulatory mechanism.

Interestingly, microbial metabolites, among which Short-Chain Fatty Acids (SCFA), like acetate, butyrate and propionate, are known to harbor the ability to alter epigenetic status of numerous cell types; studied examples thereof include the effect of such compounds on immune cells (78). In the context of human reproduction, it is conceivable that microbial metabolites, directly (e.g., via SCFA) or indirectly (e.g., acidification, alkalization, inflammatory responses), can either support (healthy microbiota) or upset (unbalanced/harmful microbiota) local cell-cell communication and tissue physiology and adaptation. As such, all relevant reproductive processes, including fertilization, implantation, placentation, immune tolerance, embryonal development, infant and adult health and may be harmfully altered by dysbiosis (167). Such gene-environment interactions are yet to be examined in detail in the context of reproductive health and disease.

### Therapeutic Opportunities

Given the association between pregnancy complications and microbiota, the question of modulation strategies is valid. Postnatal dietary strategies, including human colostrum/milk or prebiotics/probiotics, reduce morbidities in preterm infants (163). Evidence of prenatal strategies to support reproductive success and fetal health is slowly emerging: modulation of early microbiota in pregnancy shows promising effects (168). Maternal bacteria were shown to enter the gastrointestinal tract of the fetus (169) and microbiota alteration in the neonate and placenta is detectable in pregnant women receiving probiotics (170) but still under debate (14).

Recently our group published a meta-analysis on the relation between vaginal microbiota and early pregnancy development after IVF and the effect of probiotics thereon (71). It provides an overview of published studies describing long term modulation of the vaginal microbiome using *Lactobacillus*-based probiotics. Patients were often treated by Metronidazole, and followed up with a *Lactobacillus*-based probiotic. Besides lactobacillus-based probiotics, mixtures of *Lactobacillus*, *Bifidobacterium* and *Streptococcus* strains were also used in different studies (71). Further studies assessing the potential to modulate pregnancy outcomes are needed.

The notion that the neonatal immune system can be shaped by early fetal microbial colonization by inference implies that reciprocal interactions between the host and microbiota exist (171). This hypothesis is in line with the evidence that factors like PIF mediate maternal immune tolerance during pregnancy (108). Fine tuning of immune modulation is not only relevant for embryo implantation but also for defense against potentially harmful microbes. An exaggerated maternal immune response is putatively linked to preterm birth and fetal loss (172). Therefore, a novel strategy could be the maternal immune system modulation by synthetic PIF (110). Inflammatory challenge during pregnancy results in endogenous PIF expression and additional administration of synthetic PIF could prevent fetal loss.

The neonatal microbiota, mainly that of the gut, skin and oral cavity, has long been postulated to be acquired postnatally known as “postnatal microbiota seeding”. Alternatively, the hypothesis of perinatal microbiota transfer and its potential relevance to infant and adult general health is gaining attention. Given the importance of transfer of maternal microbiota to the child via colonization, the significance of birth mode is of high interest (173). Vaginal birth exposes the baby to maternal vaginal and intestinal microbiota, whereas cesarean section limits exposure of the newborn to parental dermal microbiota and any microbes present in the surgical theater. The “prophylactic” antibiotic treatment of the mother during labor or cesarean section may aggravate any effect of non-natural birth on colonization and may also negatively affect intestinal microbiota of the offspring (174). Hence, birth mode may have long-lasting effects on the composition of the newborn gut microbiome, and predispose for adverse health outcomes (174). The notion that cesarean delivery deprives the infant of exposure to vaginal microbiota and consequently leads to neonatal dysbiosis has led to the popularized, yet unsubstantiated and potentially hazardous practice of “vaginal seeding”. In a pilot study of 18 mother-infant pairs, there was partial restoration of microbiome (mainly of the skin and oral cavity and less so of the gut) in infants exposed to vaginal fluids from a vaginally placed gauze after cesarean delivery (*n* = 4), compared to non-exposed infants (*n* = 7), and resembling the microbiome of vaginally delivered infants (*n* = 7) (175). This widely cited trial was criticized for the small sample size and the potential bias from confounders such as intrapartum antibiotic prophylaxis and maternal BMI (173, 174). Although postnatal vaginal seeding altered newborn intestinal microbiota composition over several months, this intervention is believed to introduce an inherent risk of transferring pathogenic microbes (e.g., HPV, GBS) onto the newborn (176). For this reason, perinatal seeding is currently not encouraged as standard practice by the American College of Gynecology and Obstetrics (177). Whether and how vaginal seeding has any long-term beneficial health effects for the offspring requires large properly conducted studies with robust microbiome analysis.

As microbiota represents an environmental factor which affects host-microbe interactions at the epigenetic level, detailed understanding of the molecular workings of the close functional interplay between host cell systems and microbiota holds the promise that therapeutic intervention strategies can be designed for the benefit of general and reproductive health. These include the use of probiotics, beneficial microbial metabolites, rational diets and/or (ant)agonists of specific biological response pathways (162). Combined the research cited in his review define opportunities for modulation of the female reproductive tract microbiota, while harnessing its protective and immuno-regulating role during pregnancy. Such opportunities should take into consideration individual differences in microbial communities, and tailor therapeutics to different anatomical and gestational factors in an attempt to provide precision tools for reproductive health.

## Conclusion

The composition and interaction of the female reproductive tract microbiome with the host not only shape the mothers’ physiology and health during pregnancy, but also that of the fetus in accordance with the developmental origins of health and disease principles. Scientific knowledge on and insight into the properties and workings of human microbiota has increased over the last decade. However, the definition and possible implications of beneficial versus harmful microbes in physiological and pathological pregnancies is just beginning to emerge. A growing body of evidence associates stability of reproductive tract microbiota to reproductive health and maternal-fetal status during gestation, in which the interplay between microbiota and the maternal immune response takes up a prominent position. The important question of whether and how reproductive tract microbiota can be modified during and beyond pregnancy is under debate and awaits solid confirmation. The challenge for future research is to deliver standardized and validated reference methods for comparative analysis and interpretation of reproductive tract microbiomes, in order to understand their role in various clinical disorders and test the implementation of individualized therapies in large prospective trials.

## Author Contributions

SA-N and EA designed and wrote the manuscript and illustrations. LW, MSc, MSp, and JV wrote the sections of the manuscript and contributed to the interpretation of the results. SM, MM, and BK conceived the original idea, supervised the project, and edited the manuscript. All authors provided the critical feedback and contributed to the final version of the manuscript.

## Conflict of Interest

The authors declare that the research was conducted in the absence of any commercial or financial relationships that could be construed as a potential conflict of interest. The reviewer, SS, declared a past co-authorship, with one of the authors, SM, to the handling editor.
